# ErbB2-positive mammary tumors can escape PI3K-p110α loss through downregulation of the Pten tumor suppressor

**DOI:** 10.1038/onc.2017.264

**Published:** 2017-08-07

**Authors:** Alexandra M. Simond, Trisha Rao, Dongmei Zuo, Jean J. Zhao, William J. Muller

**Affiliations:** 1 Rosalind and Morris Goodman Cancer Research Centre, Department of Biochemistry, McGill University, Montreal, Quebec, Canada; 2 Departments of Cancer Biology, Dana–Farber Cancer Institute, Pathology, Harvard Medical School, Boston, Massachusetts 02115, USA

**Keywords:** Breast cancer, ErbB2, p110α, p110β, PTEN, Therapy escape

## Abstract

Breast cancer is the most common cancer among women and 30% will be diagnosed with an ErbB2-positive cancer. Forty percent of ErbB2-positive breast tumors have an activating mutation in p110α, a catalytic subunit of phosphoinositide 3-kinase (PI3K). Clinical and experimental data show that breast tumors treated with a p110α-specific inhibitor often circumvent inhibition and resume growth. To understand this mechanism of resistance, we crossed a p110α conditional (p110αflx/flx) mouse model with mice that overexpresses the ErbB2/Neu-IRES-Cre transgene (NIC) specifically in the mammary epithelium. Although mammary-specific deletion of p110α dramatically delays tumor onset, tumors eventually arise and are dependent on p110β. Through biochemical analyses we find that a proportion of p110α-deficient tumors (23%) display downregulation of the Pten tumor suppressor. We further demonstrate that loss of one allele of PTEN is sufficient to shift isoform dependency from p110α to p110β in vivo. These results provide insight into the molecular mechanism by which ErbB2-positive breast cancer escapes p110α inhibition.

## Introduction

Breast cancer is the most commonly diagnosed cancer in women worldwide, and 70% of women with breast cancer have mutations in the phosphoinositide 3-kinase (PI3K) pathway [1]. PI3K is an essential lipid kinase whose downstream effects involve cell growth, proliferation and survival [2,3,4,5]. PI3K functions by phosphorylating phosphatidylinositol-4,5-bisphosphate (PIP2) on its 3′ hydroxyl group to generate phosphatidylinositol-3, 4,5-bisphosphate (PIP3) [6,7,8]. PIP3 is an important second messenger that recruits PI3K effectors to the membrane allowing subsequent activation of the pathway. Phosphatase and TENsin homolog (PTEN) is an essential lipid phosphatase that antagonizes PI3K by dephosphorylating PIP3 and has antagonistic functions to PI3K [2,3,4,5].

PI3K represents a large family of protein kinases that is divided into three classes, of which, class I is the most commonly studied in breast cancer. Class I is further subdivided into class Ia, which are activated mainly by Receptor Tyrosine Kinases (RTKs), such as the ErbB2/ErbB3 heterodimer, and class Ib that are primarily driven by G protein-coupled receptors (GPCRs) [9,7]. Both subclasses are made up of a p110 catalytic subunit and a p85 regulatory subunit [2,10,11]. The catalytic subunit of Class I PI3K has four isoforms: p110α, p110β, p110γ and p110δ [12,13]. Both p110α and p110β are ubiquitously expressed, while p110γ and p110δ mainly expressed in leukocytes [14,15]. Today, the most studied isoform remains p110α due to its 40% mutation frequency in breast cancer and 15% mutation rate across all cancers [16,17,18]. This makes p110α the most mutated class Ia isoform [19]. However, p110β has been increasingly in the spotlight due to its association with PTEN loss, an aberration associated with hereditary cancers and frequently observed in breast cancers [20,21,22,23]. Recent publications have suggested that PTEN-null breast tumors often depend on p110β for PI3K signaling downstream of GPCRs, while PTEN wild-type tumors depend on p110α downstream of RTKs [24,25]. Genetic context also seems to influence the choice between p110α or p110β dependency in the absence of PTEN. For example, in ErbB2-positive or KRAS mutated breast cancers, PTEN-null tumors are solely dependent on p110α [26,27].

ErbB2 is an RTK that is found to be amplified and overexpressed in 20-30% of breast cancers, 40% of which have an activating mutation in p110α [28,29]. A wide variety of pan and isoform-specific inhibitors have been developed against PI3K, some of which are currently in clinical trials [2]. Pan-PI3K inhibitors have been associated with toxicity, so there have been increasing numbers of clinical trials investigating isoform-specific inhibitors [28,2]. Unfortunately, inhibition of p110α becomes ineffective over time both in vitro and in vivo, indicating the development of resistance mechanisms. [30,31]. Notably one group found that in response to a p110α-specific inhibitor, luminal breast cancer cells rapidly compensate for p110α through the engagement of p110β [30]. Another group has found that continued treatment with a p110α -specific inhibitor leads to a durable response in patients with a PIK3CA mutation, however, patients ultimately stop responding to therapy and develop lung metastasis that display PTEN-loss and p110β dependency. [32]

We have previously shown that loss of p110α in the Mouse Mammary Tumor Virus (MMTV)-ErbB2-IRES-Cre (NIC model) results in abrogation of mammary tumor development over an initial 8-month observation period [33]. Given that resistance to p110α specific inhibitors occur with time, we decided to evaluate p110α-deficient tumors over an extended period of 24-months. We find that although, ErbB2-driven mammary tumorigenesis is severely delayed in the absence of p110α, the majority of animals eventually develop tumors. To understand the mechanism by which acquired resistance was occurring in our system, we performed detailed genetic and molecular analyses of the resulting tumors. We show that one mechanism by which p110α-loss is rescued is through spontaneous Pten downregulation. We further demonstrate that reduction in PTEN levels, through the loss of one allele, is sufficient to rescue the delay in tumor onset caused by the loss of p110α, which was found to occur through the p110β isoform. The isoform switch that occurs after p110α loss raises the concern about using isoform-specific inhibitors as a way to reach durable remission in patients who have ErbB2-positive breast cancer, and allows us to suggest a therapeutic approach that is potentially more effective.

## Results

### Loss of p110α significantly delays tumor onset, and impairs tumor growth and metastasis, with a subset of tumors exhibiting downregulation of the Pten tumor suppressor

**Figure 1:**
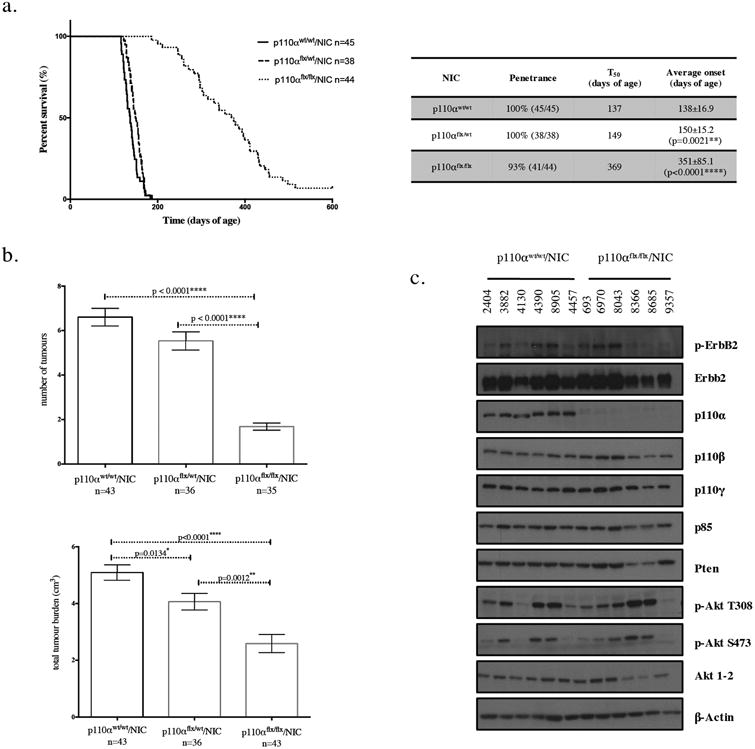
Loss of p110α significantly delays tumor onset, and impairs tumor growth and metastasis, with a subset of tumors exhibiting down regulation of the Pten tumor suppressor (a) Kaplan-Meier tumor onset curve for NIC mice that are wildtype, heterozygous, or homozygous for the p110α conditional allele. The table indicates for each genotype the penetrance (percentage of animals that developed tumours), T_50_ (age when 50% of the animals have tumours), and average tumour onset with standard deviation for each of the curves shown on the graph. p values were calculated using a two-tailed student t-test. (b) The number of tumors and total tumor burden at endpoint (5-7 weeks post-palpation). The error bars represent the standard error of the mean and the p values were calculated using a two-tailed student t-test. (c) immunoblot analysis of tumor lysates (20*μ*g) for the genotypes indicated.

To understand how ErbB2-positive tumors escape p110α inhibition, we crossed a conditional p110α mouse strain [34], to an activated ErbB2/Neu-IRES-Cre mouse model driven specifically to the mammary epithelium through the mouse mammary tumor virus promoter (MMTV) (NIC model) (supplemental figure 1a) [35]. The resulting mice have ErbB2 activation and Cre-mediated deletion of p110α in one or both of the conditional alleles (supplemental figure 1b,c). Consistent with our previous report [33], mammary specific deletion of both alleles of p110α significantly delayed tumor onset as compared to the parental strain, with an average of 351 days (n=44) versus 138 days (n=45) respectively. However, loss of one allele of p110α has a significant but limited impact on tumor onset with an average of 150 days (n=38) (figure1a). In the p110α-deficient animals that eventually developed tumors, the number of tumors (n=35) and total tumor burden (n=43) was significantly lower than in the wild-type animals (n=43) (figure 1b). The p110α-deficient tumors also exhibited reduced metastatic potential to the lungs : 72% (n=39) in the p110α-deficient strain to 45% (n=20) in the heterozygous-p110α mice versus 9% (n=23) in the wild-type animals (supplemental figure 2a). At a histological level, all tumors of each genotype displayed a classic adenocarcinoma phenotype that is typical of NIC model (supplemental figure 2b).

Given the known importance of p110α in the PI3K/PTEN/AKT signaling axis, we next evaluated the levels of key downstream components of the PI3K pathway. We find that despite the loss of p110α, tumors retain robust phosphorylation of Akt. In fact, a subset of tumors that tend to have higher levels of p-Akt are also found to downregulate Pten, ErbB2, p85 and total Akt. Of 39 p110α-deficient tumors, 9 displayed a reduction in Pten levels (23%) (supplemental figure 3). However, most tumors that have high levels of p-Akt are not found to downregulate Pten, ErbB2, p85 or total Akt, suggesting the involvement of multiple mechanisms.

These observations indicate that regardless of p110α status, tumors remain dependent on PI3K downstream signaling.

### p110α-deficient ErbB2 tumors remain dependent on PI3K

**Figure 2:**
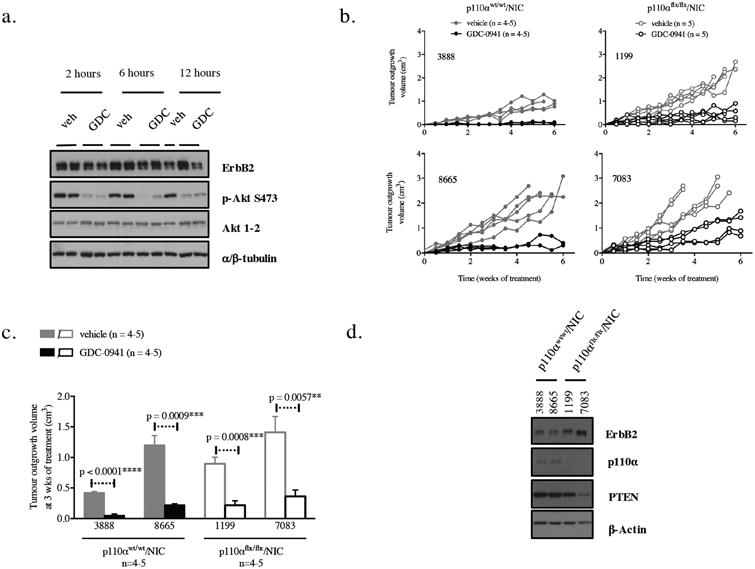
p110α-deficient ErbB2 tumors remain dependent on PI3K (a) Immunoblot of p110α^wt/wt^/NIC mammary tumor lysates (20*μ*g) from NCr mice injected with (500 000 tumor cells) after treatment with either a vehicle (veh) 125mg/kg of GDC-0941 by oral gavage for the indicated times, (b) Tumor outgrowth in NCr mice injected with tumor cells (500 000 tumor cells) of the indicated genotypes and treated with either vehicle or 125mg/kgof GDC-0941 by oral gavage daily for 6 weeks (c) Difference in tumor growth in between vehicle-treated and GDC-0941-treated tumors after 3 weeks of treatment. The p-values were calculated using a two-tailed Student t-test. (d) Immunoblot of NCr tumor lysates (20*μ*g) from the indicated genotypes.

Given the observed downregulation of PTEN in a subset of p110α-deficient/NIC tumors, we hypothesized that these tumors may still be dependent on PI3K signaling through another p110 isoform. To test this hypothesis, we determined whether p110α-deficient tumors remained sensitive to a pan-PI3K inhibitor (GDC-0941) [36]. Consistent with this contention, we found that potent inhibition of p-Akt occurred within 2 hours of treatment and is still apparent after 12 hours (figure 2a). Next, we established tumors in athymic nude mice (NCr) from two p110α-deficient and two wild-type tumor lines (n=4-5 per line), and treated these mice with either a vehicle or GDC-0941. In contrast to animals treated with vehicle, the animals treated with GDC-0941 showed drastic reduction in tumor growth (figure 2b,c).

We further show that dependency on PI3K is observed regardless of Pten status, as tumor cells having wild-type or reduced levels of PTEN were equally responsive to GDC-0941 inhibition (figure 2d). These observations indicate that a p110 isoform switch may be dependent or independent of PTEN status, suggesting the existence of at least two distinct mechanisms by which tumors escape loss of p110α. Taken together, these results indicate that, despite the loss of p110α, NIC tumor cells remain dependent on PI3K enzymatic activity.

### PTEN haploinsufficiency can compensate for loss of p110α in ErbB2 mammary tumor progression

**Figure 3:**
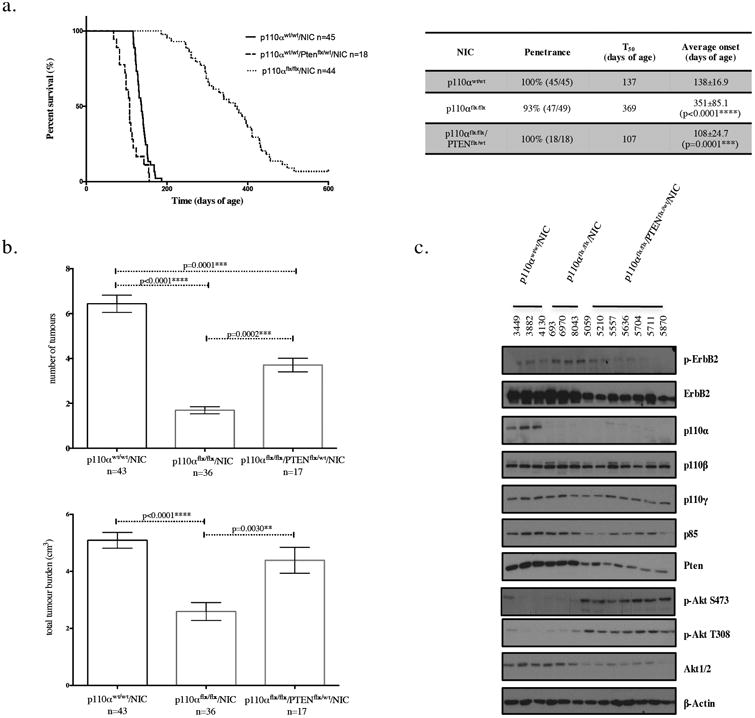
PTEN haploinsufficiency can compensate for loss of p110α in ErbB2 mammary tumor progression (a) Kaplan-Meier tumor onset curve for NIC mice that are wild-type or homozygous for the p110α conditional allele and wild type or heterozygous for the PTEN conditional allele. The table indicates for each genotype the penetrance (percentage of animals that developed tumors), T_50_ (age when 50% of the animals have tumors), and average tumor onset with standard deviation for each of the curves shown on the graph, p-values were calculated using the log-rank (Mantel-Cox) test, (b) The number of tumors and total tumor burden at endpoint (5-7 weeks post-palpation). The error bars represent the standard error of the mean and the p-values were calculated using a two-tailed student t-test (c) Immunoblot analysis of tumor lysates (20*μ*g) for the genotypes indicated.

The data presented thus far argue that at least one of the molecular mechanisms by which p110α-deficient tumors escape p110α dependency may be through Pten downregulation. To directly address this hypothesis, we crossed a conditional PTEN strain to the NIC model to generate mice with heterozygous loss of PTEN and overexpression of activated ErbB2 in the mammary epithelium (supplemental figure 4a.b.c.d). This allowed us to produce mice that will express reduced levels of Pten, similar to what we observed in a subset of p110α-deficient tumors. Consistent with the causal role of Pten downregulation in tumor escape of p110α-loss, tumor onset in p110α-deficient/NIC animals lacking an allele of PTEN was dramatically accelerated from 351 days (n=45) in the p110α-deficient/NIC animals to 108 days (n=18) but only modestly accelerated tumor onset as compared to wild-type NIC mice that had tumors appear at 138 days (n= 44) (figure 3a). The number of tumors (n=17), total tumor burden (n=17), and the incidence of lung metastasis (n=17) was increased in p110α-deficient/PTEN haploinsufficient animals as compared to the p110α-deficient strain (n=36, n=36, n=23) (Figure 3b, supplemental figure 5a). Histologically, we observed an adenocarcinoma phenotype across all genotypes (supplemental figure 5b). Most strikingly, when analyzing protein levels of PI3K signaling pathway components, we observed that tumors that have lost p110α and are PTEN-haploinsufficient have lower levels of ErbB2, p85, and total Akt, as well as higher levels of p-Akt. These results are consistent with what we observed in p110α-deficient tumors that have spontaneous Pten downregulation (figure 3c). This suggests that loss of one allele of PTEN is sufficient to shift p110 isoform dependency away from p110α in an ErbB2 mouse model, mimicking p110α inhibition, and that PTEN is indeed the driver of the p110 isoform switch.

We have previously shown that PTEN loss in the NIC model accelerates tumorigenesis, but in the presence of wild type p110α [37]. This report, along with our findings presented, emphasize the importance of PTEN levels in p110α-dependent and independent mammary tumorigenesis.

### Tumors that have lost p110α are p110β dependent

**Figure 4:**
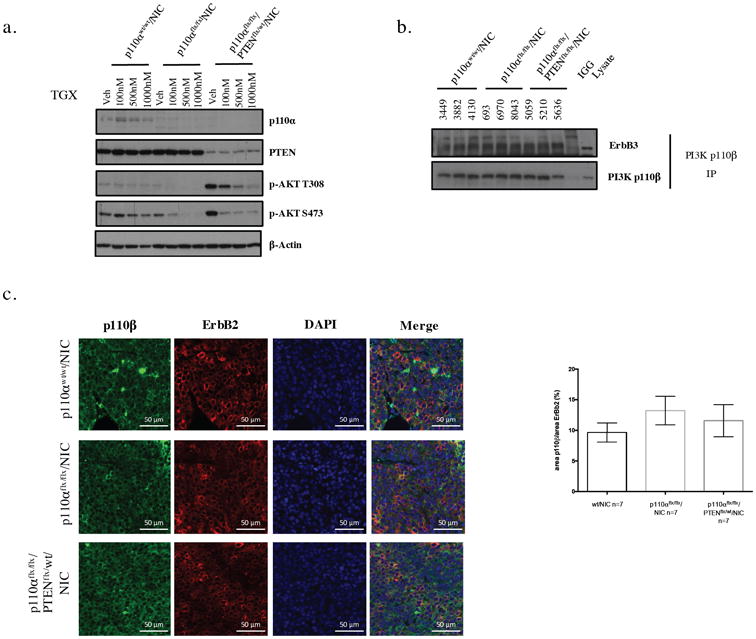
Tumors that have lost p110α are p110β dependent (a) Immunoblot of NCr cells treated with vehicle or 100nM, 500nM or 1000nM of TGX-221 (p110β3 specific inhibitor) for 6 hours, (b) Co-immunoprecipitation of p110βand ErbB3, p110βand p-85 (500*μ*g). (c) Immunoflorescence staining of p110βand DAPI on paraffin-embedded mammary tumor tissue, scale bar represents 50*μ*m.

To confirm that Pten deficiency is responsible for p110 isoform switch, we next investigated whether p110α-deficient tumor cells with PTEN-haploinsufficiency remained sensitive to p110β inhibition by treating them with a p110β-specific inhibitor (TGX221) [38]. Using Akt phosphorylation as a readout for PI3K activity, we observed that both p110α-deficient and p110α/PTEN-deficient tumors cells displayed sensitivity to p110β inhibition, whereas, wild-type tumor cells displayed constant levels of p-Akt. These results indicate that a 2-fold decrease in Pten levels can induce p110β-driven tumors (figure 4a). The fact that these p110α-deficient tumors are p110β-dependent suggests that there are multiple mechanisms involved in p110 isoform switch. Furthermore, our results indicate that p110β may be signaling downstream of receptors other than the ErbB2/ErbB3 heterodimer. Consistent with this expectation co-immunoprecipitation analyses of tumor lysates revealed that p110β associates equally with the ErbB3 receptor in all the genotypes. Because the binding of p110β to ErbB3 is not increased in response to p110α-loss, p110β may be signaling through another receptor (figure 4b). Consistent with this data, immunofluorescence analyses for p110β and ErbB2 on tumor sections, revealed similar co-localization of p110β and ErbB2 across genotypes, confirming that p110β recruitment to the ErbB2/ErbB3 heterodimer is not increased in p110α-deficient tumors (n=7 for each genotype) (figure 4c). Taken together, these data argue that PTEN downregulation is sufficient to drive the switch from p110α to p110β in an ErbB2 mouse model mimicking p110α inhibition.

## Discussion

One of the ongoing challenges in the treatment of breast cancer is the emergence of acquired resistance to therapies targeting oncogenic drivers such as PI3K and ErbB2. To uncover the molecular basis for resistance of ErbB2-positive breast cancer to p110α-specific inhibition, we used an ErbB2 mouse model mimicking p110α inhibition in the goal of understanding tumor therapy escape over time. We established that mammary epithelial-specific ablation of p110α dramatically delays tumor onset, although, the majority of animals eventually develop tumors that are no longer dependent on p110α. Using a pan-specific inhibitor we showed that all tumors remain dependent on PI3K. Interestingly, a proportion of p110α-deficient tumors display downregulation of the Pten tumor suppressor. We found that transgenic loss of an allele of PTEN is sufficient to rescue the delay in tumor onset observed and induce tumors that are dependent on p110β.

The observation that the subtle changes in Pten protein levels can have dramatic effects on p110 isoform dependency is critical as it concerns the design of targeted therapies for breast cancer. It is well known that Pten is a haploinsufficient tumor suppressor and that slight changes in its protein levels can be controlled by a variety of post-transcriptional and post-translational events [23].

In our mouse model, loss of p110α has a significant impact on ErbB2-driven mammary tumorigenesis, which fits with the theory that ErbB2-positive tumors are p110α addicted and signal through p110β as a last resort, as p110β signals less efficiently through RTK's [39,40].

**Figure 5:**
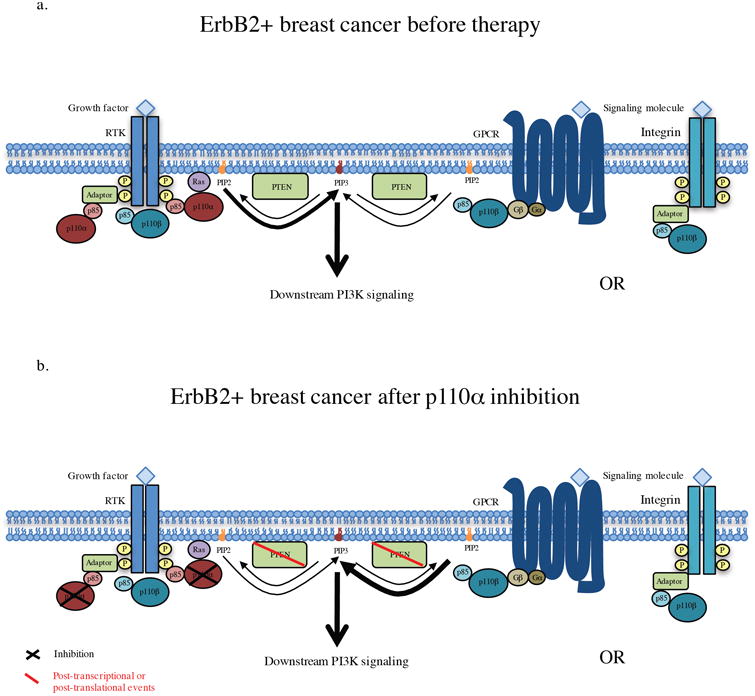
Summary diagram of P13K signaling in response to p110α targeted therapy (a) PI3K signaling in ErbB2 positive breast cancer before therapy, where the majority of PDK signaling occurs through p110α binding to the ErbB3/ErbB2 heterodimer. (b) PI3Ksignaling after treatment with a p110α-specific inhibitor. In response to p110α a subset of tumors escape through p110β in response to PTEN downregulation/heterozygous loss. In these tumors p110β may signal through theErbB2/ErbB3 heterodimer but is more likely to be signaling through another receptor, possibly GPCR's or lntegrins.

It still remains unclear why Pten has such an interconnected role with isoform specificity in ErbB2-positve breast cancer. One possible explanation is that Pten status can alter the levels of a variety of proteins some of which may be ligands to a receptor to which p110β would have more affinity to then RTKs, such as GPCRs or Integrins. Alternatively, PTEN loss may also affect p110β signaling through upregulation of GPCRs or Integrins [41,42,43]. (figure 5)

Evidence from our study that may support this point is the lower levels of p85 protein in p110α-deficient tumors with spontaneous PTEN downregulation. p110β has been found to not necessarily need p85 to bind to GPCR receptors, as it binds directly on the catalytic domain [43,44]. Consistent with this possibility, we also observed that increased p110β dependency did not correlate with increased recruitment to the ErbB3 receptor or with increased co-localization with the ErbB2/ErbB3 heterodimer. However, another explanation for the results observed in figure 4 could be that p110β is equally recruited to the ErbB2/ErbB3 heterodimer but only displays kinase activity in response to Pten downregulation.

Based on our genetic studies, we could predict that treatment of ErbB2-positive breast cancer with a p110α-specific inhibitor would lead to the development of resistance through dependency on p110β. Given that these tumors seem to solely escape through p110β is reassuring and suggests that a combinational therapy between p110α and p110β specific inhibitors may be the best solution, to this date, for during remission. There are still many interesting avenues to be discovered in the molecular mechanisms by which p110 isoform switch occurs and future studies may permit us to develop better and less toxic therapeutic approaches for ErbB2-positive patients.

## Methods

### Animal husbandry

Our animals were housed at the animal facility in the Goodman Cancer Research Center and our experiments followed the approved animal use protocol. All strains used in this study were on an FVB/N background. The strains utilized for the study were : conditional p110α[33], conditional PTEN (129/J, obtained from The Jackson Laboratory, Bar Harbor, ME) and NIC [35]. All mice used for experimental purposes were female and they were housed for a maximum of 600 days.

### Genotyping and excision PCR

Genotyping on mouse tails was performed at weaning age and at sacrifice. Excision PCR was conducted at tumor endpoint. DNA was extracted from tails and tumors as outlined later. Primers for genotyping were: p110α F: CTGTGTAGCCTAGTTTAGAGCAACCATCTA, R: CCTCTCTGAACAGTTCATGTTTGATGGTGA PTEN F: ACTCAAGGCAGGGATGAGC, R: GCCCCGATGCAATAAATATG Neu F: TTCCGGAACCCACATCAGGCC, R: GTTTCCTGCAGCAGCCTACGC Cre: F GCTTCTGTCCGTTTGCCG, R: ACTGTGTCCAGACCAGGC Primers for excision PCR were: p110α F: CTGTGTAGCCTAGTTTAGAGCAACCATCTA, R: ACAGCCAAGGCTACACAGAGAAACCCTGTC PTEN P1: ACTCAAGGCAGGGATGAGC, P2: AATCTAGGGCCTCTTGTGCC, P3: GCTTGATATCGAATTCCTGCAGC. All primers were used at a concentration of 10μM.

Taq (20120X, Qiagen, Venlo, The Netherlands) was used for p110α and PTEN genotyping and excision PCR's with twice the amount of recommended Taq was used for the excision PCR. Easy Taq (AP111, TransGen-EasyTaq, Beijing, China) was used for the Neu and the Cre genotyping PCR's

### Mammary tumor monitoring

Female nulliparous mice were monitored weekly by mammary palpation and animals with tumors were sacrificed 5-7 weeks after tumor onset. Tumors were measured using a caliper and total volume was determined with the following formula: (4/3 × (3.14159) × (length/2) × (width/2)ˆ2). For animals with multiple masses had the individual volumes of each tumor were added to determine total volume. Mice were sacrificed before the total total tumor volume reached 6cm3 or before a single tumor reached 2.5cm3

### Tissue sample processing

Tissue samples were collected at necropsy and either flash frozen in liquid nitrogen and stored at -80°C until further use, or fixed immediately in 10% neutralized formalin for 24 hours. Fixed tissue was paraffin-embedded and sectioned at a thickness of 4μm by the Histological core facility in the Goodman Cancer Research Center at Mcgill university. H&E staining was performed by the Histology Core Facility.

#### Lung metastasis analysis

Lung metastasis was assessed by counting lesions in 5 × 50 μm step sections of lungs from tumor-bearing mice.

### GDC-0941 in vivo assay

Tumor cells were isolated from Ncr tumor outgrowths (see supplemental methods for tumor dissociation protocol) and 5.0×105 tumor cells were injected into the mammary fat pad of Ncr mice (one side). Mice were treated with 125mg/kg of GDC-0941 or vehicle by oral gavage daily for 6 weeks. Tumor outgrowth was monitored bi-weekly until tumor burden endpoint (2.5 cm3)

### Protein extraction

Tumour lysates were prepared from flash frozen tumor tissue, crushed with a mortar and pestle, and lysed in PLCγ or TNE lysis buffer (see supplemental methods). Protein concentration was determined by brandford assay.

### Immunoblotting/immunoprecipitation

For immunoblot, primary antibodies used were: p-ErbB2 (cell signaling# 2249, 1:1000), ErbB2 (Santa Cruz #284, 1:1000), p110α (cell signaling #4249, 1:1000), p110β (santacruz #602, 1:1000), p110γ (cell signaling #5405, 1:1000), p85 (cell signaling #4257, 1:1000), Pten (cell signaling #9559, 1:1000), p-Akt thr308 (cell signaling #4056, 1:500), p-Akt ser473 (cell signaling #9271, 1:1000), Akt1-2 (santa-cruz #1619, 1:1000), β-actin (Sigma A5441, 1:10000). Secondary antibodies used were conjugated to horserashish peroxidase (HRP) (jackson laboratory). For immunoprecipitation, a p110β antibody (santacruz #602) was used. Primary antibodies for immunoblot were ErbB3 (santacruz #285 1:1000) and p110β (santacruz #602 1:1000)

### DNA/RNA extraction

tail DNA was extracted from tail pieces at weaning age and at sacrifice using salt precipitation (see supplementary methods). DNA was extracted from tumor tissue using phenol-chloroform (see supplemental methods) and RNA was extracted using the Qiashredder colums and the DNA/RNA mini kit from Qiagen

### qRTPCR

cDNA was generated from 1ug of tumor RNA using the M-Mulv Reverse Transcriptase (#M0253S, New England Biolabs, Ipswich, Massachusetts, United States) Oligo-dT(23VN) and a murine RNase inhibitor (New England Biolabs). Real-time PCR was performed using the Roche lightcycler master mix on a Roche lightcycler 480. Samples were always run in triplicates and normalized to Gapdh. Primers using for RTPCR: Gapdh: F : CATCAAGAAGGTGGTGAAGC, R: GGGAGTTGCTGTTGAAGTCG, p110α F: TCCATCAGCTTCTGCAAGAC R: CTTCCCTTTCTGCTTCTTGG, PTEN: F: CATTGCCTGTGTGTGGTGATA R: AGGTTTCCTCTGGTCCTGGTA

### Immunofloresence

Primary antibodies: p110β (santacruz #602, 1:1000), ErbB2 (Dako A0485) Pten (cell signaling #9559, 1:1000). Secondary antibodies: Alexa fluor-488 TSA, Alexa fluor-456 TSA, Alexa fluor-455 from thermofisher. Colocolization of p110β and ErbB2 was quantified using 6 different images from each sample. Images were obtained using the Axioscan slide scanner from Zeiss and analyzed with the Metamorph software. Slides stained for PTEN were imaged using the LSM 800 from Zeiss.

### TGX-221 in vitro assay

Mammary tumor cells were isolated from transplanted outgrowths in NCr mice at the same time and transplanted in DMEM with 5% Fetal Bovin Serum (FBS). Cells were treated 1 day after plating with 100nM, 500nM or 1000nM of TGX-221 inhibitor (final DMSO concentration of 0.2%) or with DMSO for 6 hours. Cells were then harvested and lysed with PLCγ lysis buffer for immunoblot analysis.

### Statistical analysis

All experiments on animals were done non-randomized and 9nblended. For the Kaplan-Meier curves, cohorts of 18 mice and over were used to ensure enough statistical power. The variance within each group was assessed by F-test. In figure 1b the number of tumors in the wild-type NIC group as compared to the p110αflx/flx/NIC and the p110αflx/wt/NIC compared to the p110αflx/flx/NIC had a significant P value. In Figure 2c, the F-test was statistically significant when comparing vehicle and GDC-0941 treatment in sample 8665. Finally, in Figure 3b the variance of the number of tumors per group was significant for wild-type NIC mice compared to p110αflx/flx/NIC and p110αflx/flx/PTENflx/wt/NIC mice. The statistical analysis used throughout the manuscript was a two tailed Student-t-test, apart from the Kaplan-Meier survival analysis which was done using the log-rank (Mantel-Cox) test. For all statistical tests p-values smaller than 0.05 were considered statistically significant.

## Supplementary Material

1

2

3

4

5

6

## References

[1] Hernandez-Aya LF, Gonzalez-Angulo AM (2011). Targeting the Phosphatidylinositol 3-Kinase Signaling Pathway in Breast Cancer. The Oncologist.

[2] Thorpe LM, Yuzugullu H, Zhao JJ (2015). PI3K in cancer: divergent roles of isoforms, modes of activation and therapeutic targeting. Nature Reviews Cancer.

[3] Engelman JA, Luo J, Cantley LC (2006). The evolution of phosphatidylinositol 3-kinases as regulators of growth and metabolism. http://doi.org/10.1038/nrg1879.

[4] Liu P, Cheng H, Roberts TM, Zhao JJ (2009). Targeting the phosphoinositide 3-kinase pathway in cancer.

[5] Vanhaesebroeck B, Guillermet-Guibert J, Graupera M, Bilanges B (2010). The emerging mechanisms of isoform-specific PI3K signalling.

[6] Czech MP IP2 and IP3: Complex Roles at the Cell Surface. Cell.

[7] Vanhaesebroeck B, Waterfield MD (1999). Signaling by distinct classes of phosphoinositide 3-kinases. Experimental Cell Research.

[8] Katso R, Okkenhaug K, Ahmadi K, White S, Timms J, Waterfield MD (2001). Cellular function of phosphoinositide 3-kinases: implications for development, immunity, homeostasis, and cancer. Annual Review of Cell and Developmental Biology.

[9] Geering B, Cutillas PR, Nock G, Gharbi SI, Vanhaesebroeck B (2007). Class IA phosphoinositide 3-kinases are obligate p85-p110 heterodimers. Proceedings of the National Academy of Sciences of the United States of America.

[10] Mellor P, Furber LA, Nyarko JNK, Anderson DH (2012). Multiple roles for the p85α isoform in the regulation and function of PI3K signalling and receptor trafficking. Biochem J.

[11] Cantley LC (2002). The Phosphoinositide 3-Kinase Pathway. Science.

[12] Carpenter CL, Duckworth BC, Auger KR, Cohen B, Schaffhausen BS, Cantley LC (1990). Purification and characterization of phosphoinositide 3-kinase from rat liver. Journal of Biological Chemistry.

[13] Hiles ID, Otsu M, Volinia S, Fry MJ, Gout I, Dhand R (1992). Phosphatidylinositol 3-kinase: structure and expression of the 110 kd catalytic subunit. Cell.

[14] Stoyanov B, Volinia S, Hanck T, Rubio I (1995). Cloning and characterization of a G protein-activated human phosphoinositide-3 kinase. Science.

[15] Chantry D, Vojtek A, Kashishian A, Holtzman DA, Wood C, Gray PW (1997). p110δ, a novel phosphatidylinositol 3-kinase catalytic subunit that associates with p85 and is expressed predominantly in leukocytes. Journal of Biological Chemistry.

[16] Karakas B, Bachman KE, Park BH (2006). Mutation of the PIK3CA oncogene in human cancers. British Journal of Cancer.

[17] Bellacosa A, Etro D, Neri LM (2005). Mutations of the PIK3CA gene in ovarian and breast cancer. The Women's Oncology Review.

[18] Bachman KE, Argani P, Samuels Y, Silliman N, Ptak J, Szabo S (2004). The PIK3CA gene is mutated with high frequency in human breast cancers. Cancer Biology & Therapy.

[19] Samuels Y, Wang Z, Bardelli A, Silliman N, Ptak J, Szabo S (2004). High frequency of mutations of the PIK3CA gene in human cancers. Science.

[20] Wee S, Wiederschain D, Maira SM, Loo A, Miller C, Stegmeier F (2008). PTEN-deficient cancers depend on PIK3CB. Proceedings of the National Academy of Sciences.

[21] Ni J, Liu Q, Xie S, Carlson C, Von T, Vogel K (2012). Functional characterization of an isoform-selective inhibitor of PI3K-p110β as a potential anticancer agent. Cancer Discovery.

[22] Torbett NE, Luna-Moran A, Knight ZA, Houk A, Moasser M, Weiss W (2008). A chemical screen in diverse breast cancer cell lines reveals genetic enhancers and suppressors of sensitivity to PI3K isoform-selective inhibition. Biochem J.

[23] Song MS, Salmena L, Pandolfi PP (2012). The functions and regulation of the PTEN tumour suppressor.

[24] Dbouk HA, Backer JM (2010). A beta version of life: p110β takes center stage. Oncotarget.

[25] Wang Q, Liu P, Spangle JM, Von T, Roberts TM, Lin NU (2015). PI3K - p110α mediates resistance to HER2-targeted therapy in HER2+, PTEN-deficient breast cancers. Oncogene.

[26] Schmit F, Utermark T, Zhang S, Wang Q, Von T, Roberts TM, Zhao JJ (2014). PI3K isoform dependence of PTEN-deficient tumors can be altered by the genetic context. Proceedings of the National Academy of Sciences.

[27] Wang Q, Liu P, Spangle JM, Von T, Roberts TM, Lin NU (2015). PI3K - p110α mediates resistance to HER2-targeted therapy in HER2+, PTEN-deficient breast cancers. Oncogene.

[28] Fruman DA, Rommel C (2014). PI3K and cancer: lessons, challenges and opportunities.

[29] Andrulis IL, Bull SB, Blackstein ME, Sutherland D, Mak C, Sidlofsky S (1998). neu/erbB-2 amplification identifies a poor-prognosis group of women with node-negative breast cancer. Toronto Breast Cancer Study Group. Journal of Clinical Oncology.

[30] Costa C, Ebi H, Martini M, Beausoleil SA, Faber AC, Jakubik CT (2015). Measurement of PIP 3 Levels Reveals an Unexpected Role for p110β in Early Adaptive Responses to p110α-Specific Inhibitors in Luminal Breast Cancer. Cancer Cell.

[31] Cheng H, Liu P, Ohlson C, Xu E, Symonds L, Isabella A (2015). PIK3C AH1047R-and Her2-initiated mammary tumors escape PI3K dependency by compensatory activation of MEK-ERK signaling. Oncogene.

[32] Juric D, Castel P, Griffith M, Griffith OL, Won HH, Ellis H Convergent loss of PTEN leads to clinical resistance to a PI(3)K[agr] inhibitor.

[33] Utermark T, Rao T, Cheng H, Wang Q, Lee SH, Wang ZC (2012). The p110α and p110β isoforms of PI3K play divergent roles in mammary gland development and tumorigenesis. Genes & Development.

[34] Zhao JJ, Cheng H, Jia S, Wang L, Gjoerup OV, Mikami A, Roberts TM (2006). The p110α isoform of PI3K is essential for proper growth factor signaling and oncogenic transformation. Proceedings of the National Academy of Sciences.

[35] Ursini Siegel J, Hardy WR, Zuo D, Lam SH, Sanguin Gendreau V, Cardiff RD (2008). ShcA signalling is essential for tumour progression in mouse models of human breast cancer. The EMBO Journal.

[36] Folkes AJ, Ahmadi K, Alderton WK, Alix S, Baker SJ, Box G (2008). The identification of 2-(1 H-indazol-4-yl)-6-(4-methanesulfonyl-piperazin-1-ylmethyl)-4-morpholin-4-yl-thieno [3, 2-d] pyrimidine (GDC-0941) as a potent, selective, orally bioavailable inhibitor of class I PI3 kinase for the treatment of cancer. Journal of Medicinal Chemistry.

[37] Dourdin N, Schade B, Lesurf R, Hallett M, Munn RJ, Cardiff RD, Muller WJ (2008). Phosphatase and tensin homologue deleted on chromosome 10 deficiency accelerates tumor induction in a mouse model of ErbB-2 mammary tumorigenesis. Cancer Research.

[38] Jackson SP, Schoenwaelder SM, Goncalves I, Nesbitt WS, Yap CL, Wright CE (2005). PI 3-kinase p110β: a new target for antithrombotic therapy. Nature Medicine.

[39] Beeton CA, Chance EM, Foukas LC, Shepherd PR (2000). Comparison of the kinetic properties of the lipid-and protein-kinase activities of the p110α and p110β catalytic subunits of class-Ia phosphoinositide 3-kinases. Biochem J.

[40] Meier TI, Cook JA, Thomas JE, Radding JA, Horn C, Lingaraj T, Smith MC (2004). Cloning, expression, purification, and characterization of the human Class Ia phosphoinositide 3-kinase isoforms. Protein Expression and Purification.

[41] Martin V, Guillermet-Guibert J, Chicanne G, Cabou C, Jandrot-Perrus M, Plantavid M (2010). Deletion of the p110β isoform of phosphoinositide 3-kinase in platelets reveals its central role in Akt activation and thrombus formation in vitro and in vivo. Blood.

[42] Schoenwaelder SM, Ono A, Nesbitt WS, Lim J, Jarman K, Jackson SP (2010). Phosphoinositide 3-kinase p110β regulates integrin αIIbβ3 avidity and the cellular transmission of contractile forces. Journal of Biological Chemistry.

[43] Dbouk HA, Vadas O, Shymanets A, Burke JE, Salamon RS, Khalil BD (2012). G Protein–Coupled Receptor–Mediated Activation of p110β by Gβγ Is Required for Cellular Transformation and Invasiveness. Science Signaling.

[44] Dbouk HA (2015). PI3King the right partner: Unique interactions and signaling by p110β. Postdoc Journal: a Journal of Postdoctoral Research and Postdoctoral Affairs.

