# Supplementary figures and images for: ErbB2-positive mammary tumors can escape PI3K-p110α loss through downregulation of the Pten tumor suppressor
Source: Oncogene. Author manuscript; Available in PMC 2018 Feb 13. (PMC5808977; doi:10.1038/onc.2017.264)

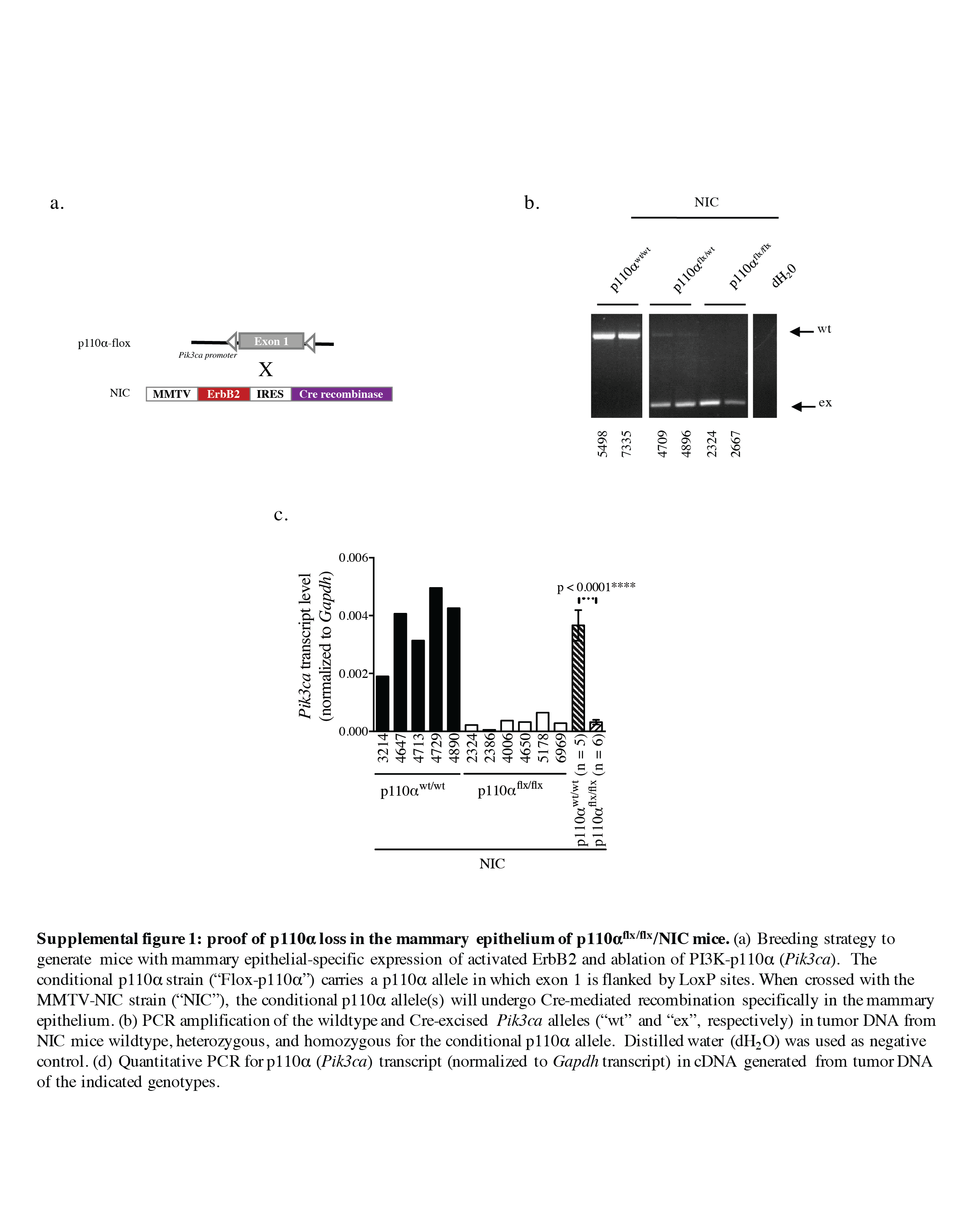

Supplement: 1 [file NIHMS888482-supplement-1.png]

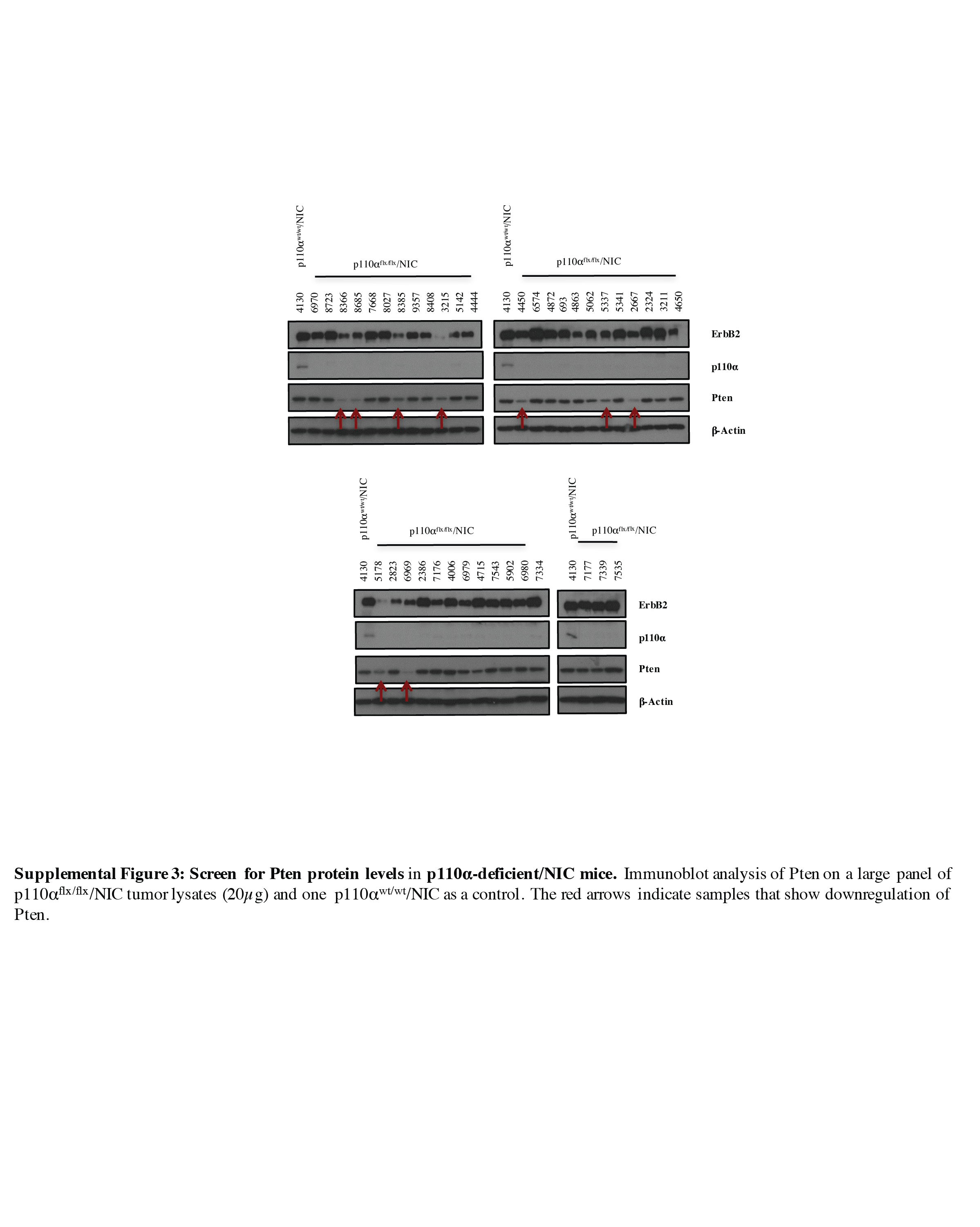

Supplement: 2 [file NIHMS888482-supplement-2.png]

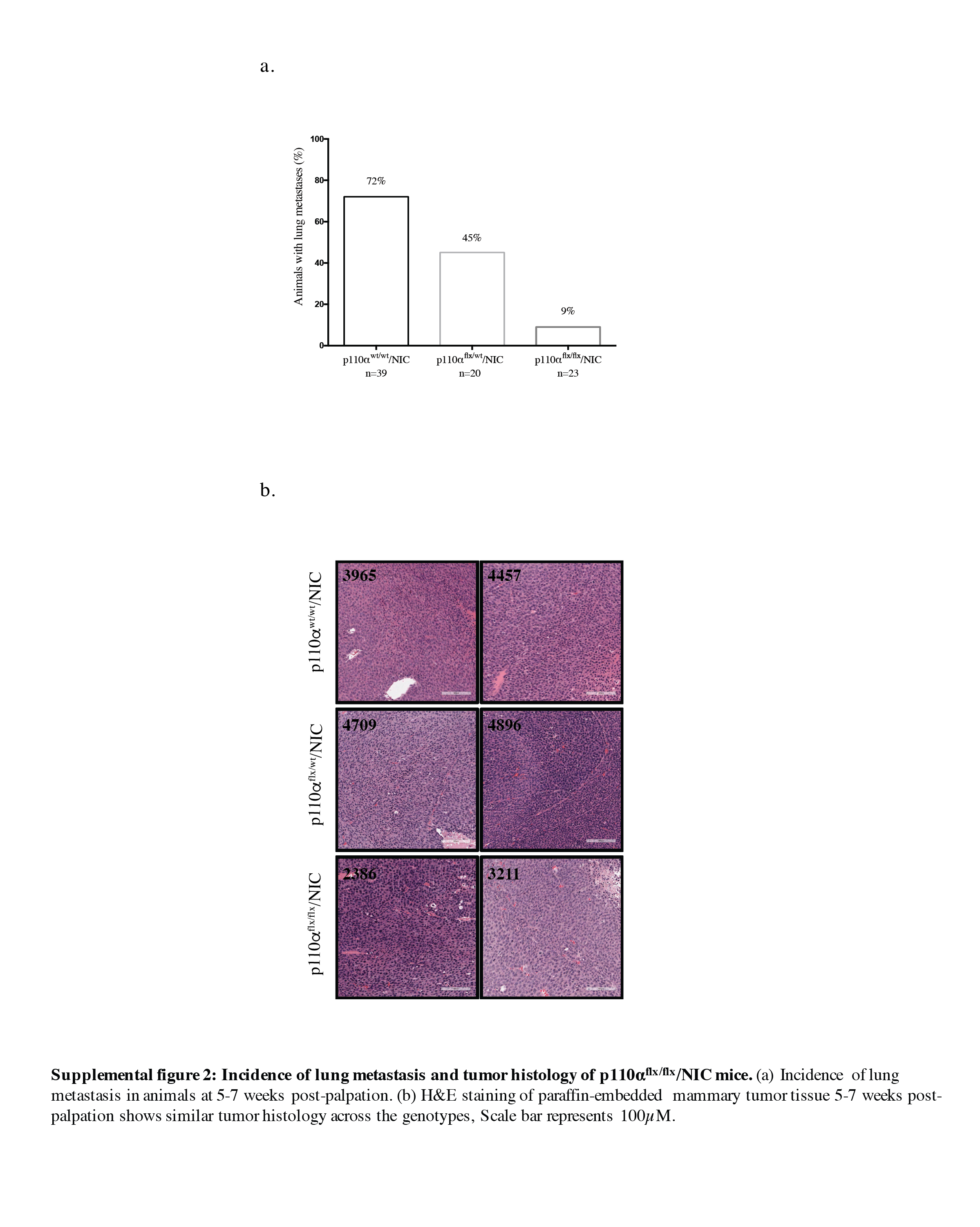

Supplement: 3 [file NIHMS888482-supplement-3.png]

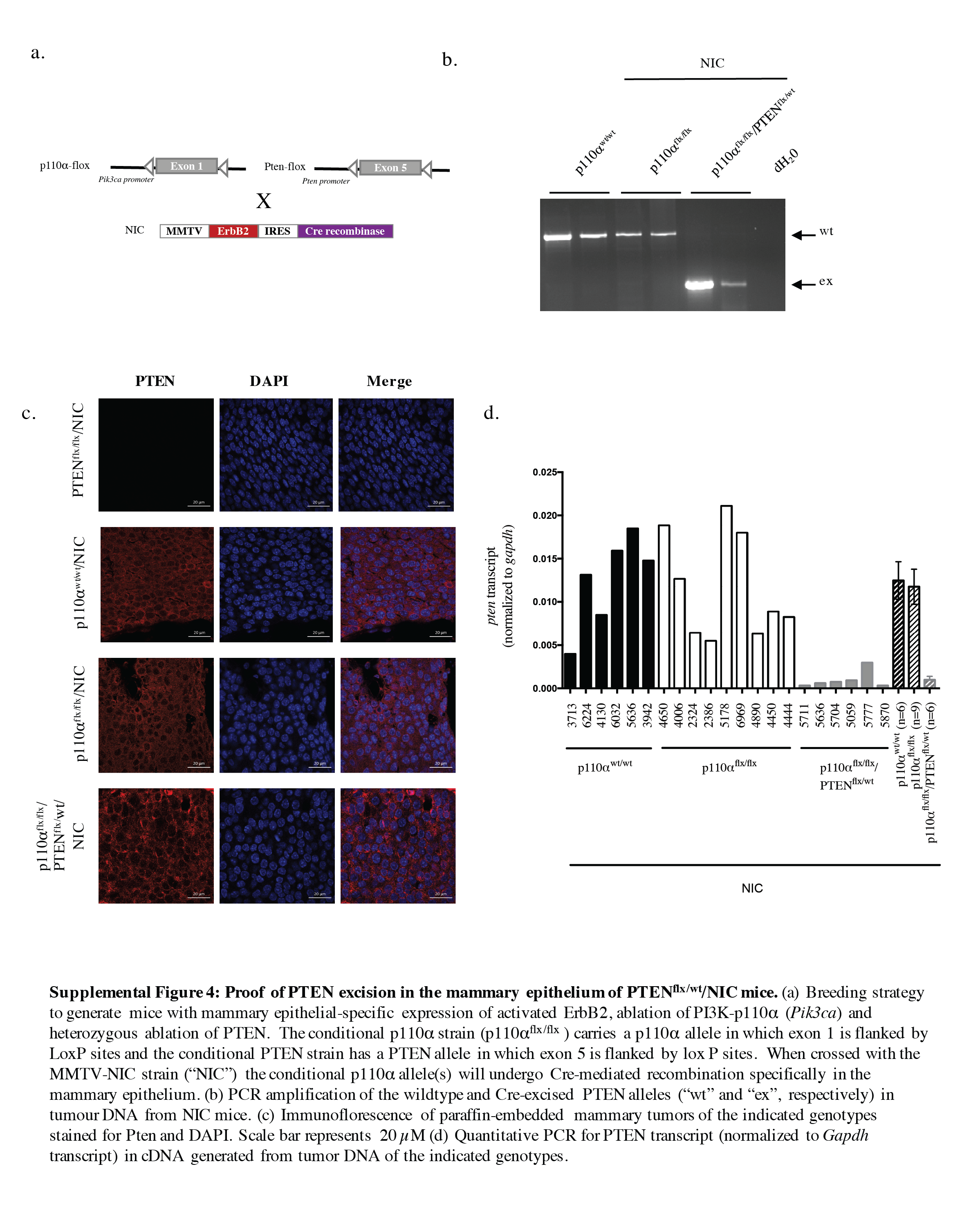

Supplement: 4 [file NIHMS888482-supplement-4.png]

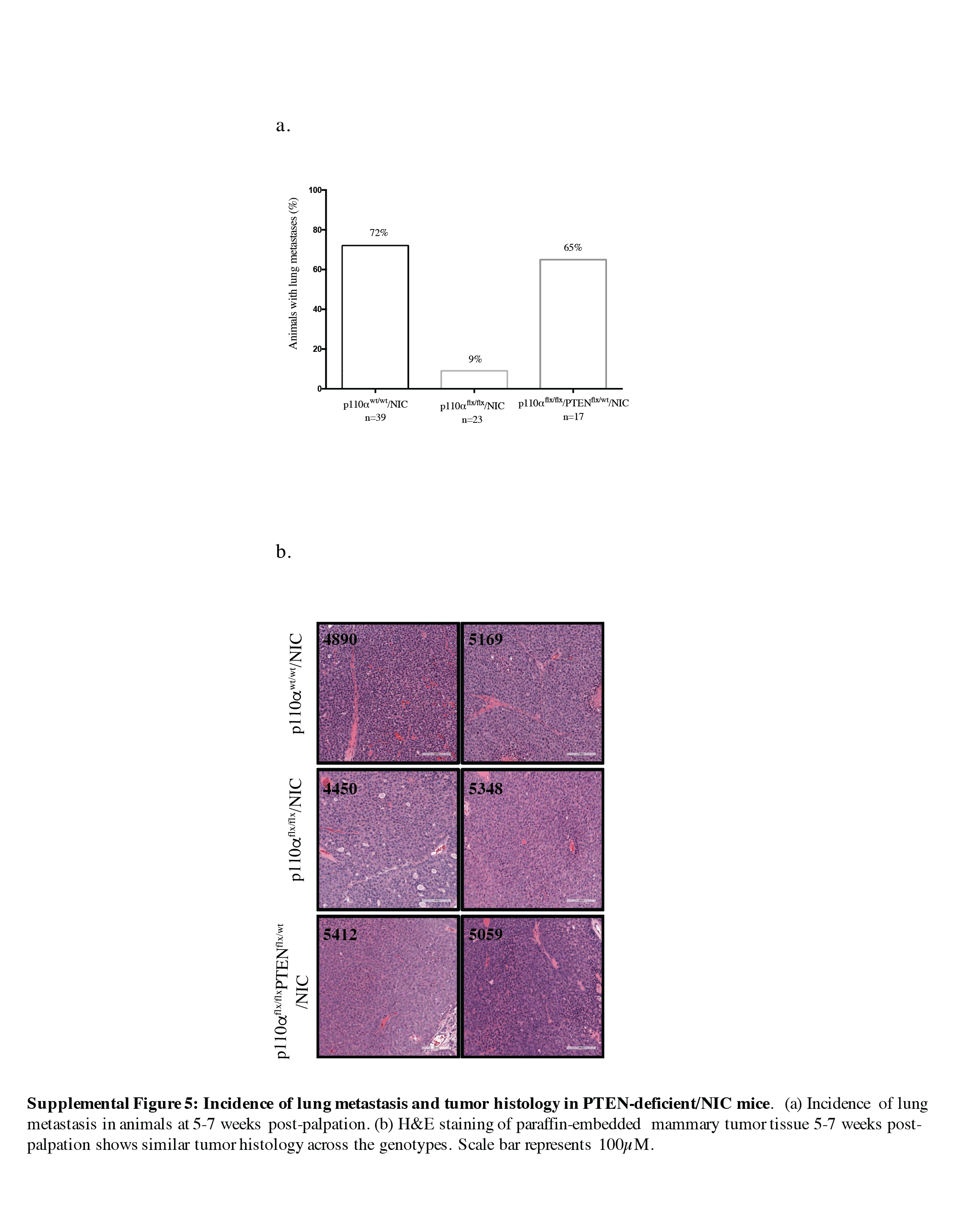

Supplement: 5 [file NIHMS888482-supplement-5.png]
