# Supplementary material for: ErbB2-positive mammary tumors can escape PI3K-p110α loss through downregulation of the Pten tumor suppressor
Source: Oncogene. Author manuscript; Available in PMC 2018 Feb 13. (PMC5808977; doi:10.1038/onc.2017.264)
Supplement: 6 [file NIHMS888482-supplement-6.docx]

**Supplementary Methods:**

Genotyping and excision PCR programs:

PCR Programs:**p110α, Neu** and **Cre** genotyping: 94ºC for 1 minute (94ºC for 30 seconds, 56ºC for 30 seconds, 72ºC for 1 minute) for 34 cycles, 72ºC for 5 minutes. **PTEN** genotyping: 94ºC for 5 minutes (94ºC for 30 seconds, 60ºC for 1 minute, 72ºC for 1 minute) for 34 cycles, 72ºC for 2 minutes.**p110α** excision: 94ºC for 1 minute (94ºC for 30 seconds, 57ºC for 30 seconds, 72ºC for 1 minute) for 34 cycles, 72ºC for 5 minutes. **PTEN** excision: 94ºC for 2 minutes (94ºC for 30 seconds, 64ºC for 1 minute, 72ºC for 1,5 minute) for 34 cycles, 72ºC for 10 minutes.

Protein extraction and Western blot:

For immunoblot analysis, samples were lysed in PLCγ lysis buffer (50 mM HEPES, 150 mM NaCl, 10% glycerol, 1% Triton X-100, 1 mM EGTA, 1.5 mM MgCl2, 10 mM NaF, 10 mM sodium pyrophosphate) . For the immunoprecipitation, samples were lysed in TNE buffer ( 50mM Tris-HCL PH=8, 150mM NaCl, 1% NP-40, 10mM NaF, 10mM sodium pyrophosphate, 2mM EDTA) both containing 1 g/mL leupeptin, 1 μg/mL aprotinin, and 1 mM Na3VO4 ) was used and all samples were incubated on ice for 1 hour. Protein lysates were then centrifuged at 13,000 rpm for 10 minutes at 4ºC and supernatant was transferred to a new tube. Protein concentration was determined by Bradford assay.

Immunoblotting:

Protein lysates were diluted to 20 µg in 6X SDS-PAGE running buffer (0.375M Tris pH 6.8, 12% SDS, 60% glycerol, 0.6M DTT, 0.06% bromophenol blue) and boiled for 10 minutes. SDS-PAGE was performed using 8% acrylamide gel and transferred onto a PVDF membrane. Membranes were washed in TBS-T (150 mM NaCl, 20 mM Tris-HCl [pH 7.5], 0.05% Tween- 20) 3 times for 5 minutes. Blocking buffer (5% BSA made up in dH2O) was then added to the membrane for 1 hour at room temperature and primary antibodies, made up in TBS-T and 5% BSA, were incubated overnight at 4ºC on a shaker. The next day, after washing 3 times for 5 minutes in TBS-T membranes were incubated with horseradish peroxidase (HRP)-conjugated secondary antibody (1:10000) for 1 hour at room temperature and membranes were washed 3 times for 5 minutes in TBS-T and incubated with ECL detection reagent and directly exposed to film. In some experiments, membranes were then washed again 3 times for 5 minutes in TBT-T and 3 more times for 5 minutes in dH2O and incubated with stripping buffer (100mM Glycine in dH2O) [pH 2] for 1h at room temperature. After stripping membranes were washed again 3 times for 5 minutes in dH2O and 3 times for 5 minutes in TBS-T, then blocked and re-probed.

Immunoprecipitation:

Protein lysates were diluted to 500µg in 500ul and were incubated with 5µl of p110β santa cruz # 602 (1µg) antibody overnight at 4ºC with rotation. Rabbit immunoglobulin cell signaling #2729 (1µg) was used as a control. 50µl of beads (PureProteome protein G magnetic beads- Millipore). per sample were pooled and washed with 1 ml of TNE lysis buffer 3 times. Each time the tube with the beads was placed on a magnetic stand and the lysis buffer was aspirated. 50µl of beads were aliquoted to each sample and incubated at 4ºC with rotation for 2 hours. Each sample was then washed with 1 ml of lysis buffer 5 times. After the last wash 30ul of 2X SDS-Page loading buffer (0.125M Tris pH 6.8, 4% SDS, 20% glycerol, 0.2M DTT, 0.02% bromophenol blue) with β-mercaptoethanol (60ul/ml) was added to each sample and they were boiled at 95ºC for 10 minutes. Samples were then analyzed by SDS-PAGE and immunoblot as described.

Mammary tumor dissociation:

Mammary tumors were excised from Ncr mice and left on ice in PBS (137mM NaCl, 2.7mM KCl, 10mM Na2PHO4, 1.8mM KH2PO4) for 2 hours. Each tumor was finely chopped with the McIlwain tissue chopper and dissociated at 37ºC for 2h in 10ml of digestion media: 10ml DMEM, 24mg collagenase B, 24mg Diaspase II, 1% penicillin-streptomycin. Dissociated tumors were centrifuged at 800rpm for 3 minutes, pellet was resuspended in 8ml of Ack lysis buffer (NH4Cl, 10 mM KHCO3, 0.1 mM EDTA) to remove the presence of any red blood cells. Samples were then centrifuged at 800rpm for 3 minutes. Pellets were resuspended in 8ml PBS containing 2mM of EDTA and spun at 800 rpm for 3minutes. Pellets were then resuspended in 8ml of PBS and spun at 800rpm for 3 minutes and ressuspened in the appropriate amount of DMEM containing 5% Fetal Bovine Serum (FBS) and plated.

DNA extraction:

Tail or tumor tissue was submerged in 500ul of tail buffer (10 mM Tris, 100 mM NaCl, 10 mM EDTA,0.5% SDS) containing 10ul of 20mg/ml proteinase K overnight in a 55ºC water bath. Tail DNA we extracted to sodium chloride: 200ul 5M NaCl was added to each tube and centrifuged at room temperature at 13000rpm for 5 minutes. The supernatant was transferred to a new tube and 1 volume of 100% ethanol was added. Tubes were then spun at 13000rpm at 4ºC for 10 minutes. All the remaining liquid was aspirated and the tubes were allowed to air dry. Once the tubes were dry, 400ul of Tris-EDTA (TE) buffer (10 mM Tris, 0.5 mM EDTA, pH 7.8) was added.

Tumor DNA we extracted using phenol-chloroform: 500ul of phenol-chloroform was added to each tube and centrifuged at room temperature at 13000rpm for 5 minutes the supernatant aqueous phase was transferred to a new tube and 500ul of chloroform was added, samples were centrifuged at room temperature at 13000rpm for 5 minutes. Then samples were added 100% ethanol and spun at 4ºC for 10 minutes. Supernatant was aspirated and tubes were submerged with 70% ethanol and centrifuged again at 13000 rpm for 10 minutes at 4ºC, supernatant was aspirated and the tubes were allowed to air dry. Once dry, 200ul of TE buffer was added.

QRTPCR:

Programs:

**Gapdh:** annealing: 56ºC, extension: 8 seconds, detection: 75ºC. **p110α:** annealing: 60ºC, extension: 8 seconds, detection: 82ºC. **PTEN:** annealing: 62ºC, extension: 8 seconds, detection: 76ºC. Each program ran for 35 cycles.

Immunofluorescence :

Slides were first deparaffinized by washing 3 times in xylenes for 3 minutes, 2 times in 100% ethanol for 2 minutes and once in 70% ethanol for 2 minutes. Next, slides were put under running tap water for 5 minutes and dH2O for 2 minutes. The antigen retrieval step was done by submerging slides in sodium citrate (10mM sodium citrate made up in dH2O) [pH 6]) and putting them in a pressure cooker for 10 minutes on high pressure. Slides were cooled 1 hour at room temperature. The slides were washed again in dH2O for 5 minutes and 2 times in PBS (137mM NaCl, 2.7mM KCl, 10mM Na_2_PHO_4_, 1.8mM KH_2_PO_4_) for 5 minutes. Sections were then blocked for 5 minutes with 1X blocking power block (Biogenex) and incubated with a primary antibody made up in 2.5% BSA in PBS for 1h at room temperature. One drop of ImmPress HRP reagent from Vector was then added to the sections for 30 minutes. Tyraminde signal amplification (TSA) 488 from Thermo Fisher Scientific was made up as described in their protocol and incubated at room temperature for 10 minutes. Slides were then washed in PBS and put through exactly the same steps starting from the antigen retrival step. The second primary antibody was then added and incubated at 4ºC overnight. For the PTEN stain, slides were washed then incubated with a secondary antibody (Alexa 555) at room temperature for 1h or for the p110β and ErbB2 stain was incubated with TSA 568 for 10 minutes. For both experiments DAPI was the added (2ul in 5ml of dH2O) for 15minutes and slides were then directly mounted.
